# An Objective Estimation of Air-Bone-Gap in Cochlear Implant Recipients with Residual Hearing Using Electrocochleography

**DOI:** 10.3389/fnins.2017.00210

**Published:** 2017-04-18

**Authors:** Kanthaiah Koka, Aniket A. Saoji, Joseph Attias, Leonid M. Litvak

**Affiliations:** ^1^Research and Technology, Advanced BionicsValencia, CA, USA; ^2^Schneider Children's Medical Center of Israel and Rabin Medical CenterPetach Tikva, Israel

**Keywords:** cochlear implant, electrocochleography, cochlear microphonic, air conduction, bone conduction, air-bone gap

## Abstract

Although, cochlear implants (CI) traditionally have been used to treat individuals with bilateral profound sensorineural hearing loss, a recent trend is to implant individuals with residual low-frequency hearing. Notably, many of these individuals demonstrate an air-bone gap (ABG) in low-frequency, pure-tone thresholds following implantation. An ABG is the difference between audiometric thresholds measured using air conduction (AC) and bone conduction (BC) stimulation. Although, behavioral AC thresholds are straightforward to assess, BC thresholds can be difficult to measure in individuals with severe-to-profound hearing loss because of vibrotactile responses to high-level, low-frequency stimulation and the potential contribution of hearing in the contralateral ear. Because of these technical barriers to measuring behavioral BC thresholds in implanted patients with residual hearing, it would be helpful to have an objective method for determining ABG. This study evaluated an innovative technique for measuring electrocochleographic (ECochG) responses using the cochlear microphonic (CM) response to assess AC and BC thresholds in implanted patients with residual hearing. Results showed high correlations between CM thresholds and behavioral audiograms for AC and BC conditions, thereby demonstrating the feasibility of using ECochG as an objective tool for quantifying ABG in CI recipients.

## Introduction

Cochlear implants traditionally have been used to treat individuals with bilateral profound sensorineural hearing loss. However, given the evolution of electrode and signal-processing technology and improved surgical techniques, individuals with low-frequency residual hearing also are able to experience benefit from a cochlear implant (Balkany et al., 2006; Fraysse et al., 2006). Moreover, by combining electrical and acoustic stimulation (EAS), benefit exceeds that of using a hearing aid or a cochlear implant alone (Von Ilberg et al., 1999; Turner et al., 2008).

In order to benefit optimally from EAS technologies, residual hearing in these subjects must be preserved. However, at least 50% of subjects lose their residual hearing after surgery (James et al., 2005; Balkany et al., 2006; Brown et al., 2010; Lenarz et al., 2013; Roland et al., 2016). The loss of residual hearing is attributed mainly to, direct trauma to the basilar membrane (Roland and Wright, 2006; Li et al., 2007) and not due to any potential interference produced by the presence of the electrode in the cochlea(Donnelly et al., 2009; Huber et al., 2010; Greene et al., 2015; Banakis et al., 2016). But several researchers have reported increased air-bone gaps (ABG) post-operatively in a subset of subjects after cochlear implantation despite using surgical techniques to reduce trauma (Attias et al., 2012; Chole et al., 2014; Raveh et al., 2014; Mattingly et al., 2016), thus suggesting that conductive components may be involved that can be attributed to the changes in middle ear mechanics and/or the presence of electrode in the cochlea.

**Figure 1:**
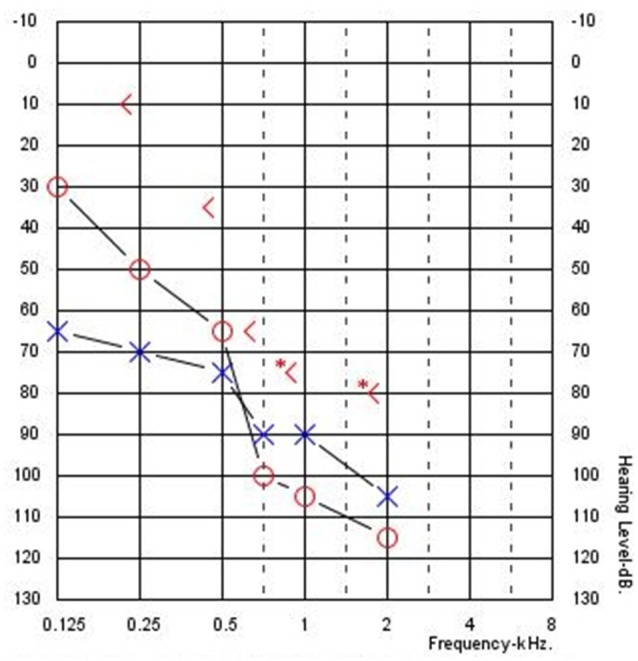
**Typical audiogram for a cochlear implant recipient with residual hearing and an air-bone gap (Subject CI04)**.

Figure 1 shows an example audiogram from a CI recipient with residual hearing showing large ABGs. These ABGs are difficult to quantify because post-operative hearing sensitivity is exclusively measured with air-conduction (AC) thresholds because bone-conduction (BC) thresholds are technically difficult to assess in individuals with severe-to-profound hearing loss. Specifically, high levels of bone oscillator stimulation in the low frequencies can result in vibrotactile sensations mistakenly reported as audible, thereby contributing to a false increase in ABG. Also, due to smaller transcranial attenuation unmasked BC thresholds may be measured due to the stimulation of the cochlea in the non-test ear. Typically, the contralateral ear is masked with a band of noise to facilitate measurement of BC thresholds in the test ear. However, limited hearing in the contralateral ear may limit the ability to measure masked BC thresholds and lead to inaccurate ABG measurement. Also, it is not possible to accurately measure BC thresholds in patients and children who cannot provide accurate responses to BC stimulation.

Because of these technical barriers to evaluating behavioral BC thresholds, it would be helpful to have an objective method to measure AC and BC thresholds for estimating ABG in implant patients with residual hearing. Koka et al. (2016) and Abbas et al. (2017) used the intra-cochlear electrodes from the implant array to measure electrocochleography (ECochG) in patients with residual hearing. Different electrical potentials such as cochlear microphonics (CM), compound action potential (CAP), summating potential (SP), and auditory nerve neurophonics (ANN) together constitute ECochG responses. The CM represents the combination of transducer currents primarily through the outer hair cell stereocilia (Dallos, 1973) and is known to follow the fine structure of the stimulus waveform. The ANN is assumed to reflect the phase locking activity of the auditory nerve fibers (Snyder and Schreiner, 1984; Lichtenhan et al., 2013; Fitzpatrick et al., 2014; Forgues et al., 2014). The CAP is generated by the auditory nerve in response to the onset and offset of the acoustic stimulus, and the SP is the direct current part of the response with multiple generators. The present study focuses on the alternating current components of the ongoing, or steady state, response to tones. The difference response, which is the difference between alternating polarities, emphasizes responses at odd harmonics of the tone frequency, which are those components of the response that change with stimulus phase. Thus, the difference response is dominated by the CM, but also includes the largest part of the ANN that is periodic with the signal. Current study focused mainly on CM responses. The summation response, which is the summation of alternating polarities, emphasizes responses at even harmonics of the tone frequency, and include the components of the response that do not change with stimulus phase. Thus, the summation response includes the asymmetric distortions present in the CM and ANN. Because these distortions are greater in the ANN than the CM, the ongoing component of the summation response can be dominated by the ANN, when it is present. However, this part of the ANN is only the distortions, and so is smaller than the part that appears in the difference response. That there is some ANN present in the difference response was shown by Forgues et al. (2014), who demonstrated a decrease in difference response by introducing a neurotoxin used to suppress auditory nerve response.

This study extends the (Koka et al., 2016) study to evaluate whether CM (which is the difference response) can be used to estimate BC thresholds in implanted patients with residual hearing. Because CM necessarily rules out any vibrotactile responses and contributions from the contralateral ear, it may be applicable for estimating BC thresholds at low frequencies. Thus, this study assessed CM responses for AC and BC stimuli in cochlear implant recipients with residual hearing.

## Methods

### Subjects

Four implant recipients with HiRes 90 K® cochlear implants (Advanced Bionics LLC, Valencia, CA) and HiFocus MidScala® electrode arrays with residual hearing participated in this study. Table 1 shows their ages and duration of implant use. The subjects were recruited based on observation of ABG with CIs. The pre-op ABGs were not available to the authors as part of this study. The etiology of the hearing loss is unknown for this group. All subjects provided written informed consent prior to participation. The study protocol (#20121035) was approved by the Western Institutional Review Board (WIRB).

**Table 1:** **Subject demographics**.

**Subject id**	**Implant type**	**Electrode type**	**Implant usage (years))**	**Age (years)**
CI04R	HiRes90K Advantage	MidScala	2	59
CI13	HiRes90K Advantage	MidScala	0.5	61
CI25	HiRes90K Advantage	MidScala	0.25	54
CI20	HiRes90K Advantage	MidScala	0.16	66

### Equipment

The AC and BC stimulus delivery and measurement system for assessing behavioral thresholds and ECochG responses was the same as that described in Koka et al. (2016). The Bionic Ear Data Collection System (BEDCS) research software of Advanced Bionics was used to control stimulus delivery and ECochG measurement. The acoustic stimuli were generated by an NI DAQ system (NI DAQ 6216, National Instruments Corporation„ Austin, TX) along with an audio amplifier (Sony PHA-2, Sony Corporation, New York, NY, USA) and presented through a ER-3A insert earphone (Etymotic Research, Inc., Elk Grove Village, IL USA) for AC and through a B-71 bone vibrator for BC. The AC and BC levels were calibrated according to ANSI S3.6 Specifications for Audiometers using clinical audiometric calibration services provided by Audiometrics (Arcadia, CA, USA). ECochG was measured using an Advanced Bionics Clinical Programming Interface (CPI-II), Platinum Series Sound Processor (PSP), and Universal Headpiece (HP). The CPI-II delivered an external trigger to synchronize acoustic/bone vibration stimulus generation and ECochG measurement through the implant.

### Pure tone audiometry and tympanometry procedures

Behavioral AC and BC pure-tone thresholds were measured at 125, 250, 500, 750, 1,000, 1,500, and 2,000 Hz using a stimulus duration of 200 ms and a step size of 2 dB using equipment described above. For each test frequency, thresholds were assessed using an ascending and descending track. The initial stimulus level for the ascending track was below the subject's audible threshold, whereas the initial stimulus level for the descending track was above the subject's behavioral threshold. The final threshold was defined as the average of the ascending and descending values. Masking was used for estimating bone conduction thresholds. Any response reported as vibrotactile or questionably vibrotactile was considered as no response.

Tympanometry was used to understand the condition of middle ear and to rule out conductive hearing loss (GSI Tympstar, Grason-Stadler Inc, Eden Prairie, MN 55344).

### Ecochg recording procedure

ECochG stimuli consisted of 50-ms tone bursts with ramp duration of 5 ms (Hanning window) presented at each subject's most comfortable level (MCL). For each frequency (125, 250, 500, 750, 1,000, 1,500, and 2,000 Hz), ECochG responses were recorded using 240 presentations with alternating polarity (120 rarefaction and 120 condensation). From the responses to alternating polarities, the difference response (CM) was extracted.

The most apical electrode contact (electrode 1) was used as the active electrode and the ring electrode, located on the electrode lead outside of the cochlea, served as the return electrode. The amplifier on the HiRes90 k implant was configured to have a gain of 1,000 and its output was digitized (9-bits) at 9,280 samples/s. The low-pass filter cutoff was set to 5,000 Hz. With these settings, the Advanced Bionics implant offers a relatively long recording window of 54.4 ms, enabling ECochG recording for low-frequency stimuli down to 125 Hz.

### Control experiments

ECochG recordings can be affected by the stimulus artifact. The bone vibrator contains a relatively strong electromagnet. It is possible that the energy generated by the electromagnet may be coupled to the implant electronics. Two control experiments were conducted to identify and quantify any artifacts that may have occurred.

First, BC ECochG waveforms were compared between stimuli delivered when the ear canal was occluded (foam plug insertion) and unoccluded. The assumption here was that due to occlusion effect, the ECochG will be increased when ear canal was occluded. The absence of stimulus artifacts was confirmed when larger ECochG responses were observed for the occluded condition compared to the unoccluded condition. These control measurements were made for all subjects.

Second, ECochG recordings were made with the bone vibrator placed close, but not touching the mastoid, to determine if any direct electromagnetic coupling occurred. A custom-built holder was used to hold the bone vibrator close to the mastoid consistently across subjects. A template subtraction technique was used to remove electromagnetic coupling artifacts from the ECochG responses if they were detected when the bone vibrator was not touching the mastoid.

### Data analysis

CM response waveforms elicited separately by AC and BC stimulation were obtained from the rarefaction and condensation waveforms by subtracting alternating polarities and computing the average. Fast Fourier Transform (FFT) analysis estimated amplitudes for each stimulus level. CM thresholds were estimated by comparing the amplitude at each stimulus level with a constant noise floor, which was constant across all subjects. Finally, CM thresholds were compared with behavioral AC and BC acoustic thresholds to determine if a correlation existed.

## Results

### Behavioral air-bone gaps (ABG)

All the subjects in the study demonstrated behaviorally-based ABGs despite tympanometry indicating normal middle ear function. The ABGs varied between 14 to 59 dB with a mean of 36 dB.

### Ecochg responses for BC

**Figure 2:**
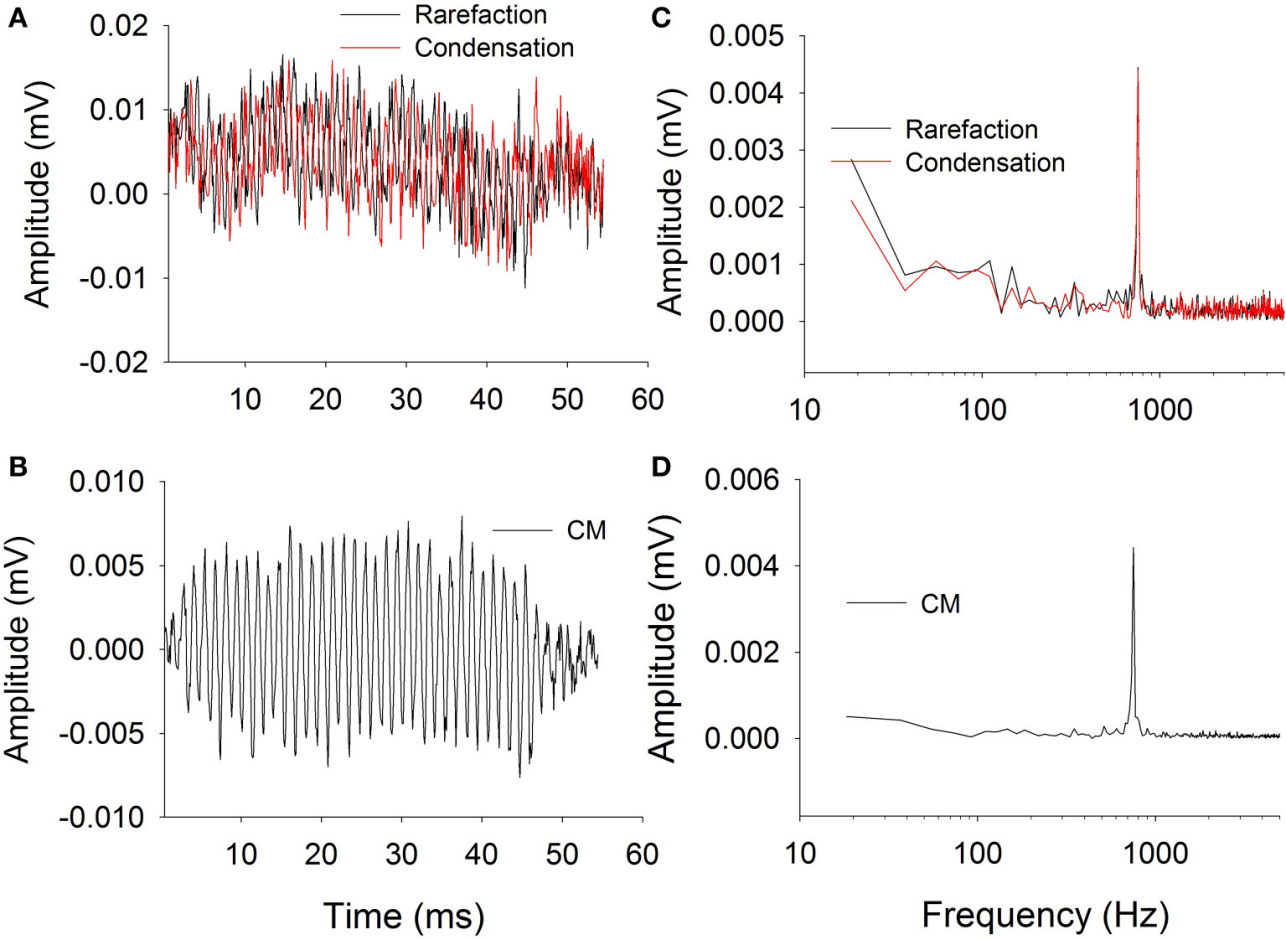
**Electrocochleography waveforms recorded with bone vibrator stimulation**. **(A)**: Raw waveforms recorded for alternating polarity stimulation. **(B)**: Difference CM response obtained by subtracting responses between alternating polarities. **(C)**: Frequency spectra of the responses to alternating polarities. **(D)**: Frequency spectrum of the difference CM response.

**Figure 3:**
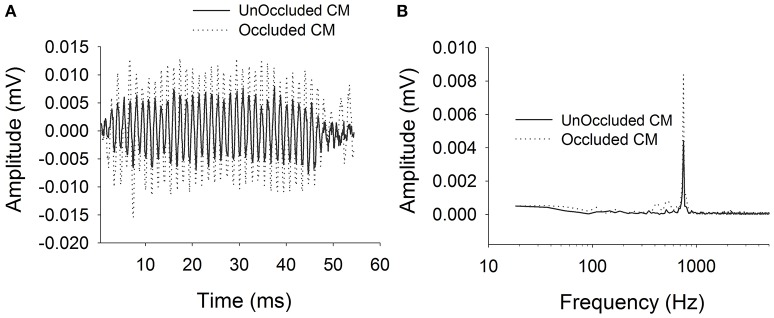
**CM responses for bone vibrator stimulation with and without occluded ear canal**. **(A)**: Time domain waveforms. **(B)**: Frequency spectra.

Figure 2 shows typical ECochG waveforms in response to BC stimulation of 750 Hz at 50 dB HL (subject CI25). The upper plots show the raw waveforms for rarefaction and condensation stimulation in the time domain (Figure 2A) and frequency domain (Figure 2C). The lower plots show in the time (Figure 2B) and frequency domains (Figure 2D) the difference waveforms computed from the responses to the alternating polarity stimuli. These responses were recorded with an occluded ear for which the subject reported an increase in loudness. Figure 3 shows CM responses from the same subject for an occluded and unoccluded ear in the time domain (Figure 3A) and frequency domain (Figure 3B). The occluded ear responses clearly show a 6 dB, doubling of amplitude compared to the unoccluded ear.

### Control experiments

No electromagnetic artifacts were observed for these subjects when stimulus levels did not elicit a vibrotactile response. Nonetheless, direct coupling electromagnetic artifacts were observed when stimulus levels were above vibrotactile thresholds, thereby indicating that artifacts exist at high levels. The template subtraction technique removed the stimulus artifact contamination at high levels.

### Ecochg vs. behavioral thresholds (AC and BC)

**Figure 4:**
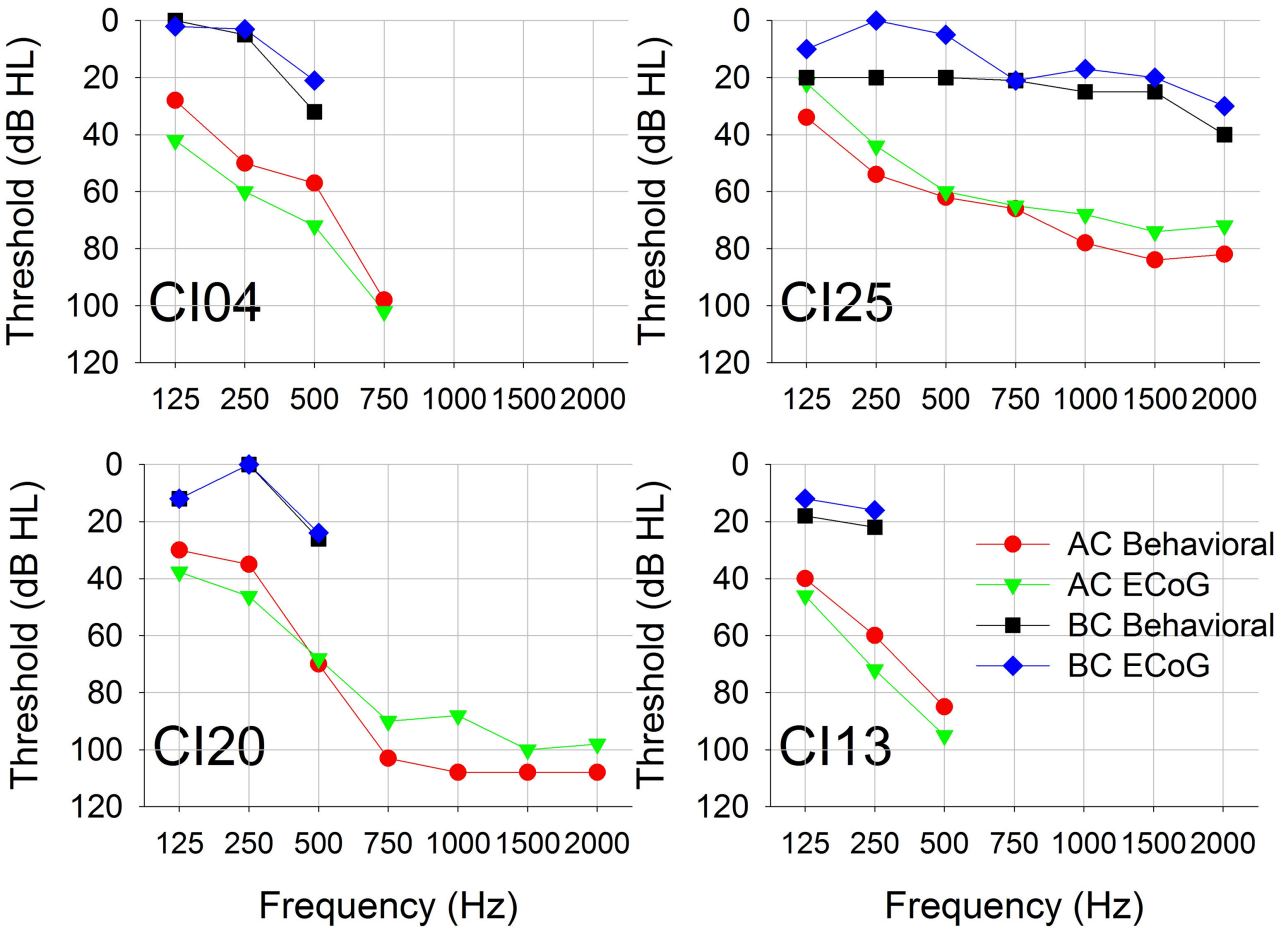
**Pure-tone AC and BC thresholds measured using conventional behavioral audiometry and ECochG**. Each panel represents data from a single subject.

Figure 4 shows behavioral and CM thresholds for all frequencies for which hearing was measureable. For all four subjects, the CM threshold profiles followed the behavioral audiometric threshold profiles. The mean and standard deviation of the difference between audiometric and CM thresholds for AC across all frequencies was −9 (±5) dB. The difference between audiometric and CM thresholds for BC across all frequencies was 6 (±6) dB.

**Figure 5:**
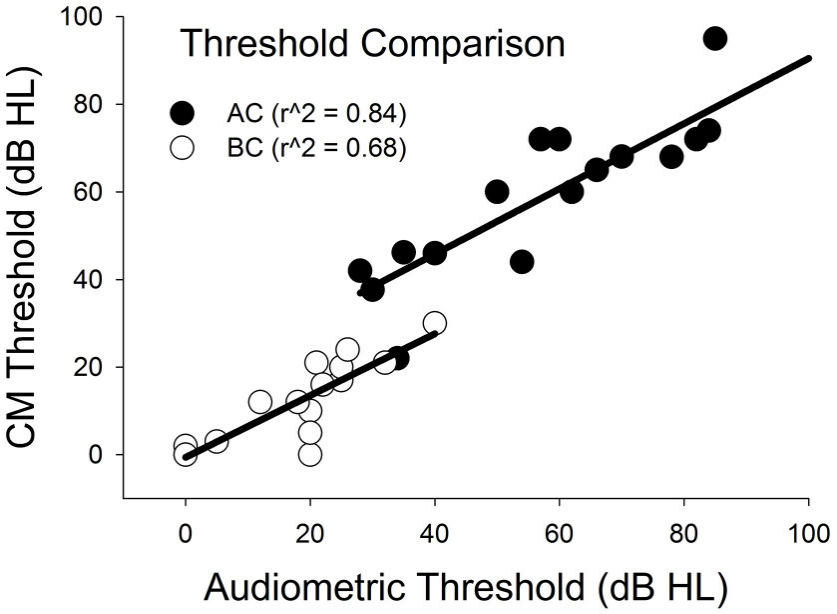
**Comparison of CM vs. audiometric thresholds across all subjects and across all frequencies for AC and BC stimulation**.

Figure 5 plots CM thresholds as a function of audiometric thresholds for both AC and BC. The correlation between CM and audiometric thresholds is highly significant across all frequencies for both AC and BC (r^∧^2 = 0.84, *n* = 21, *p* < 0.001 for AC; r^∧^2 = 0.68, *n* = 15, *p* < 0.001 for BC). The ABG for behavioral responses was 36 (±12) dB and for CM thresholds was 43 (±12) dB. There was no significant difference between ABG measured using audiometry or ECochG (*p* = 0.115, *n* = 15).

## Discussion

This study measured pure-tone audiometric thresholds and CM thresholds for AC and BC stimulation in four implanted individuals with residual hearing. Across the range of test frequencies, behavioral sensitivity and CM thresholds were highly correlated for both AC and BC stimulation. Moreover, the ABG estimated by the ECochG responses provided a reliable surrogate for behavioral ABG in these subjects.

These results are similar to Koka et al. (2016) for AC thresholds and to Abbas et al. (2017) who showed that CM thresholds approximated behavioral AC thresholds better than auditory nerve neurophonics or compound action potential thresholds. Unique to this study is the demonstration that ECochG responses to BC stimulation can provide an objective indicator of BC thresholds that are not corrupted by vibrotactile responses and does not require contralateral masking. One caveat is that care should be taken to limit BC vibrator output so as not to create electromagnetic artifacts at high stimulus levels. These results suggest that ECochG can be used as an objective tool to verify behavioral BC thresholds in CI patients with residual hearing and ABG. In implant patients, intra-cochlear electrode is used to measure ECochG which simplifies the measurement of evoked potentials especially in pediatric patients.

With the observation that ABG may exist after implantation of patients with residual hearing (Chole et al., 2014; Raveh et al., 2014; Mattingly et al., 2016) and in normal-hearing animals after implantation (Hod et al., 2016), this ECochG method can provide an objective tool to estimate reliable ABG without technical issues of measuring behavioral BC thresholds in CI subjects. The fact that this group of subjects had ABG in the presence of normal tympanometry suggests that the ABG originated in the inner ear rather than the middle ear. On the other hand acute studies looking at effect of electrode in the cochlea did show only less than 5 dB differences between air and bone conduction in temporal bones (Donnelly et al., 2009; Huber et al., 2010; Greene et al., 2015; Banakis et al., 2016). Quesnel et al. (2016) suggested that the changes in residual hearing after initial preservation may be due to intracochlear fibrosis and new bone formation changing the compliance of round window and not due to degeneration of hair cells. The current EcochG measurement may acts a tool to monitor ABG chronically and understand whether the increased ABG is due to chronic changes in the cochlea.

## Conclusion

ECochG responses can provide an objective method for estimating ABG in cochlear implant recipients with residual hearing in the implanted ear.

## Author contributions

Authors equally contributed to conception and design, drafting the article; and final approval of the version to be published and KK and AS contributed for acquisition of data, analysis and interpretation of data.

### Conflict of interest statement

The authors declare that the research was conducted in the absence of any commercial or financial relationships that could be construed as a potential conflict of interest.
